# Cardiac adaptations from 4 weeks of intensity-controlled vigorous exercise are lost after a similar period of detraining

**DOI:** 10.14814/phy2.12302

**Published:** 2015-02-23

**Authors:** Cheryl D Waring, Beverley J Henning, Andrew J Smith, Bernardo Nadal-Ginard, Daniele Torella, Georgina M Ellison

**Affiliations:** 1 Stem Cell and Regenerative Biology Unit (BioStem), Research Institute for Sport and Exercise Sciences, Liverpool John Moores UniversityLiverpool, L3 3AF, UK; 2 Centre of Human & Aerospace Physiological Sciences and Centre for Stem Cells & Regenerative Medicine, Faculty of Medicine & Life Sciences, King's College LondonLondon, SE1 1UL, UK; 3 Laboratory of Molecular and Cellular Cardiology, Department of Medical and Surgical Sciences, Magna Graecia UniversityCatanzaro, 88100, Italy

**Keywords:** Cardiac stem/progenitor cells, detraining, physiological remodeling

## Abstract

Intensity-controlled (relative to VO_2max_) treadmill exercise training in adult rats results in the activation and ensuing differentiation of endogenous c-kit^pos^ cardiac stem/progenitor cells (eCSCs) into newly formed cardiomyocytes and capillaries. Whether these training-induced adaptations persist following detraining is undetermined. Twelve male Wistar rats (∼230 g) were exercised at 80–85% of their VO_2max_ for 30 min day^−1^, 4 days week^−1^ for 4 weeks (TR;*n* = 6), followed by 4 weeks of detraining (DTR; *n* = 6). Twelve untrained rats acted as controls (CTRL). Exercise training significantly enhanced VO_2max_ (11.34 mL kg^−1^ min^−1^) and wet heart weight (29%) above CTRL (*P* < 0.05). Echocardiography revealed that exercise training increased LV mass (∼32%), posterior and septal wall thickness (∼15%), ejection fraction and fractional shortening (∼10%) compared to CTRL (*P* < 0.05). Cardiomyocyte diameter (17.9 ± 0.1 *μ*m vs. 14.9 ± 0.6 *μ*m), newly formed (BrdU^pos^/Ki67^pos^) cardiomyocytes (7.2 ± 1.3%/1.9 ± 0.7% vs. 0.2 ± 0.1%/0.1 ± 0.1%), total cardiomyocyte number (45.6 ± 0.6 × 10^6^ vs. 42.5 ± 0.4 × 10^6^), c-kit^pos^ eCSC number (884 ± 112 per 10^6^ cardiomyocytes vs. 482 ± 132 per 10^6^ cardiomyocytes), and capillary density (4123 ± 227 per mm^2^ vs. 2117 ± 118 per mm^2^) were significantly greater in the LV of trained animals (*P* < 0.05) than CTRL. Detraining removed the stimulus for c-kit^pos^ eCSC activation (640 ± 98 per 10^6^ cardiomyocytes) and resultant cardiomyocyte hyperplasia (0.4 ± 0.3% BrdU^pos^/0.2 ± 0.2% Ki67^pos^ cardiomyocytes). Capillary density (3673 ± 374 per mm^2^) and total myocyte number (44.7 ± 0.5 × 10^6^) remained elevated following detraining, but cardiomyocyte hypertrophy (15.0 ± 0.4 *μ*m) was lost, resulting in a reduction of anatomical (wall thickness ∼4%; LV mass ∼10% and cardiac mass ∼8%, above CTRL) and functional (EF & FS ∼2% above CTRL) parameters gained through exercise training. These findings demonstrate that cardiac adaptations, produced by 4 weeks of intensity-controlled exercise training are lost after a similar period of detraining.

## Introduction

The beneficial effects of a program of exercise are well documented. Failure to spend 15–30 min per day on gentle exercise such as brisk walking has been reported to increase the risk of developing heart disease by 20–30% and shortens the life span by 3–5 years (Wen and Wu 2012). In addition to being a preventative measure, exercise is prescribed as part of rehabilitation in the treatment of heart disease (Shephard and Balady 1999; Guiraud et al. 2012; Oldridge 2012; Ingle and Carroll 2013).

During a program of exercise training, the heart undergoes physiological ventricular remodeling to adapt to the added stress resulting in increased cardiac mass, function, and contractility (Jin et al. 2000; Weeks and McMullen 2011; Ellison et al. 2012). This occurs at least in part through hypertrophy of individual cardiomyocytes (Kemi et al. 2002, 2004, 2005). However, we and others have recently shown that new cardiomyocyte formation contributes to this process (Boström et al. 2010; Waring et al. 2014). We demonstrated that following intensity-controlled exercise training there is an activation of the endogenous, c-kit^pos^ CD45^neg^ cardiac stem/progenitor cell (eCSC) compartment, which express transcription factors indicative of their commitment and ensuing differentiation into the cardiomyocyte and endothelial lineages (Waring et al. 2014). This significant eCSC expansion and differentiation coincided temporally and quantitatively with the progressive increase in newly formed cardiomyocytes and capillaries, and therefore strongly supports a precursor–product relationship between the c-kit^pos^ eCSCs and the newly formed, differentiated cardiac cell progeny (Waring et al. 2014).

In contrast to the physiological adaptation of the heart in response to exercise training, the effects of exercise cessation or detraining are less understood. In 2000, Mujika and Padilla defined detraining as the partial or complete loss of training-induced adaptations in response to an insufficient stimulus. Currently, the effects of a period of detraining on exercise-induced ventricular remodeling remains unclear with some groups advocating that training-induced adaptations persist (Marini et al. 2008; Carneiro-Júnior et al. 2010; Lehnen et al. 2010), whereas others report complete loss (Kemi et al. 2004; Bocalini et al. 2010; Weiner et al. 2012; Carneiro-Júnior et al. 2013) after detraining. The reasons for these discrepancies may be due to (1) different types of exercise stimulus used; (2) different durations of the training and detraining periods; or (3) variation in the parameters investigated, with some studies assessing changes at a cellular level, whereas others focus on changes at an organ or functional level, with very few investigating both.

Here, using our previously established treadmill exercise protocol in rats, which is intensity controlled according to individual VO_2max_, (Waring et al. 2014), we determine the effects of detraining on exercise-induced ventricular remodeling, assessing changes at a cellular level (cardiomyocyte hypertrophy, c-kit^pos^ eCSC activation, and new cardiomyocyte and capillary formation), through to anatomical changes at the organ level (cardiac hypertrophy, LV wall thickness, and LV internal diameter) through to functional/physiological changes (VO_2max_, EF, and FS).

## Methods

### Ethical approval

All experimental procedures were performed in accordance with the British Home Office Animals (Scientific Procedures) Act 1986 by appropriately qualified staff.

### Animals, exercise training, and testing

A total of 24 adult male Wistar rats (∼230 g, ∼10 weeks of age) were used for this study. Animals were purchased from Harlan (UK), housed in groups of four animals/cage and maintained throughout the whole study on a 16 h/8 h light/dark cycle at a temperature of 19–23°C and a relative humidity of 55 ± 5% with food and water available ad libitum. Upon arrival at the Life Sciences Support Unit, animals were acclimatized to their new surroundings and staff performing the study over a 2 week period. After this all animals were familiarized with motorized treadmill running and maximal oxygen uptake (VO_2max_) testing was performed as previously described (Waring et al. 2014). Following a 2 week familiarization period, all animals were allowed to rest for 2 days before maximal oxygen uptake (VO_2max_) was determined for each individual rat. VO_2max_ tests were performed using the Oxymax gas analyzing system for small animals (Columbus Instruments). The volume of the air supplied was 4.5 L min^−1^ and the gas analyzer was calibrated with a reference gas mixture before each test. The VO_2max_ test protocol involved stepwise increases of the treadmill speed as follows: after a 3 min period of acclimatization (6 m min^−1^), the treadmill was then started at 10 m min^−1^, and the speed was incrementally increased 3 m min^−1^ every 2 min until 22 m min^−1^ after which the gradient of the treadmill was increased by 5° every 2 min until O_2_ consumption reached a plateau and the rat reached exhaustion. The highest VO_2max_ measured at each workload was taken as a measure of each rat's running economy (VO_2submax_) for that workload, and at the last step, as VO_2max_. Animals were encouraged to run by tapping the front and side of the chamber and then using small electrical stimuli that produced no more than a momentary (<1 sec), mild buzzing sensation (maximum of six stimuli per rat). The electrical current produced (min 25 VAC 0.34 mA, max 120 VAC 1.6 mA) does not exceed that sufficient to produce a buzzing sensation when touched by the human finger.

Animals were randomly assigned to one of four groups, 4 week or 8 week control (CTRL), exercise trained (TR) or a detrained (DTR) group (*n* = 6 per group). CTRL animals received no exercise training, whereas TR and DTR animals were exercised for 30 min day^−1^, 4 days week^−1^ for up to 4 weeks at 80–85% of their individual VO_2max_. With the exception of the 30 min exercise training period, CTRL animals were treated exactly the same as TR and DTR animals and cages contained a mixture of both CTRL and TR/DTR animals. After 2 weeks of exercise training, VO_2max_ was reassessed on exercising animals and treadmill speed and incline were adjusted to ensure animals were still performing at 80–85% of their individual VO_2max_. VO_2max_ was measured again on 4 week CTRL and TR animals at the end of the 4 week training period, prior to sacrifice and tissue collection. For the following 4 weeks, 8 week CTRL and DTR animals remained unexercised before final VO_2max_ tests were performed at 8 weeks. To track new cell generation, Bromodeoxyuridine (BrdU; MP Biomedicals, Cambridge, UK) (40 mg kg^−1^ body weight) was administered intraperitonally (i.p) twice daily throughout the 4 week training period to TR and 4 week CTRL animals and then during the following 4 week detraining period only, to 8 week CTRL and DTR animals. The 8-week CTRL and DTR animals did not receive BrdU during the training period.

### Assessment of cardiac function

In vivo echocardiography was performed using a Vivid q ultrasound system (GE Healthcare, Hatfield, UK) on 6 TR animals after 4 weeks of exercise training and re-assessed at 8 weeks following the 4 week period of detraining (DTR group). Echocardiography was also performed on the six age-matched CTRLs at 4 and 8 weeks. In brief, animals were anesthetized with the minimum amount of inhaled isoflurane needed to prevent movement and placed in the supine position. Hair was removed from the chest and LV images were obtained using a 12L-RS transducer (5.0–13.0 MHz) placed parasternally. Interventricular septum thickness (SWT), posterior wall thickness (PWT), and LV internal diameter (LVD) were measured in diastole (D) and systole (S) using M-mode echocardiography at the level of the papillary muscle. Data of 3–5 consecutive heart cycles were recorded digitally and analyzed using Echo Pac software (GE Healthcare). LV ejection fraction (EF), fractional shortening (FS), and LV mass were all estimated using formulas previously validated in small animal models (Litwin et al. 1995; Reffelmann and Kloner 2003; Kim et al. 2011). EF = (LVEDD^2^ − LVESD^2^)/LVEDD^2^, FS = ((LVEDD − LVESD)/LVEDD)*100 and LV mass = 1.04*((SWTD + PWTD + LVEDD)^3^ − (LVEDD^3^)).

### Animal sacrifice and tissue processing

Animals were killed by cervical dislocation and hearts were arrested in diastole using a 0.1 mol L^−1^ cadmium chloride solution (Sigma, Gillingham, UK) before removal from the chest cavity. Whole hearts were weighed, washed briefly in PBS before 24 h fixation in formalin (Sigma) then stored for 72 h in 70% ethanol. The atria were removed and the ventricles dissected into the apex, mid, and base regions and all tissue was processed for paraffin embedding using a Leica TP1020 tissue processor as previously described (Waring et al. 2014). Five micrometer tissue sections were cut using a Leica RM2235 microtome, mounted onto polysine microscope slides (ThermoFisher, Loughborough, UK), and stored at room temperature until processing for immunohistochemistry.

### Cardiomyocyte hypertrophy and number

Cardiomyocyte diameter was assessed in 4 and 8 week CTRL, TR, and DTR animals (*n* = 6 per group). Five micrometer tissue sections were treated with hematoxylin (Sigma) and eosin (Sigma) solutions to identify nuclei and cytoplasm, respectively, and the transverse diameter at the level of the nucleus was measured for 60 cardiomyocytes/animal at ×20 magnification using a Nikon ECLIPSE E1000M light microscope and Lucia G software (LUCIA G version 4.81; Laboratory Imaging Ltd, Prague, CZ). LV cardiomyocyte number was determined, as previously described (Torella et al. 2004; Waring et al. 2014).

### Immunohistochemistry and quantification

BrdU incorporation was assessed using the BrdU detection kit (Roche, West Sussex, UK) and cardiomyocytes were identified by costaining for alpha sarcomeric actin (*α*-sarc: Sigma). Nuclei were identified by DAPI. Tissue sections were also stained for Ki67 (Abcam, Cambridge, UK), *α*-sarc, and DAPI to identify cycling cardiomyocytes at time of sacrifice. For all four groups, 60 random fields at ×100 magnification were counted/animal and the number of BrdU^pos^ and Ki67^pos^ cardiomyocytes calculated as a % of total cardiomyocytes counted. To assess capillary density, sections were treated with an antibody against the endothelial marker von Willebrand Factor (vWF, Millipore, Watford, UK) and counterstained with hematoxylin to detect nuclei. Microvessels with a circumference spanning 1–3 endothelial cells were counted as capillaries (Ellison et al. 2011; Waring et al. 2014). For each animal, capillary density was assessed in 20 random fields at ×40 magnification and represented as capillary number per mm^2^. To determine c-kit^pos^ CD45^neg^ eCSC numbers, sections were double stained for c-kit (R&D Systems, Abingdon, UK) and CD45 (Santa Cruz Biotechnology, Insight Biotechnology Ltd, Middlesex, UK). The number of c-kit^pos^ CD45^neg^ nuclei were counted in 30 random fields per animal at ×40 magnification and expressed as number per 10^6^ cardiomyocytes. Nuclei were counterstained with DAPI and cardiomyocytes were identified by staining for *α*-sarc.

Cardiomyocyte diameter, BrdU^pos^ and Ki67^pos^ cardiomyocyte number, capillary density, and c-kit^pos^ CD45^neg^ eCSC number were assessed for the mid region of the left ventricle only, as we previously found no significant differences for these variables between the apex, mid, and base regions (Waring et al. 2014). Immunofluorescence staining was imaged using an LSM 710 scanning confocal microscope and Zen 2009 software (Zeiss, Cambridge, UK). All analysis was performed by investigators who were blind to the group assignment.

### Statistical analysis

All results are presented as Mean ± SD. Significance was determined by the analysis of variance (ANOVA) using the statistical software package SigmaPlot version 12.0 (Systat software Inc, Chicago, IL) and all tests were two sided. The Holm–Sidak post hoc method was used to locate the differences. Significance was set at *P* < 0.05.

## Results

### Exercise-induced improvements in aerobic exercise capacity, physiological remodeling, and cardiac function are lost following a period of detraining

The 4 week CTRL and TR groups began the study with similar body weights (*P* > 0.05; 4 week CTRL, 240 ± 8 g vs. TR, 240 ± 5 g) and maximal aerobic exercise capacity, as measured by VO_2max_ (*P* > 0.05; 4 week CTRL 52 ± 3 mL kg^−1^ min^−1^ vs. TR 52 ± 4 mL kg^−1^ min^−1^). Likewise, the 8 week CTRL and DTR groups showed no significant differences in body weight or VO_2max_ at baseline (*P* > 0.05; body weight, 8 week CTRL 256 ± 8 g vs. DTR 255 ± 4 g; VO_2max_, 8 week CTRL 51 ± 4 mL kg^−1^ min^−1^ vs. DTR 52 ± 4 mL kg^−1^ min^−1^).

**Figure 1:**
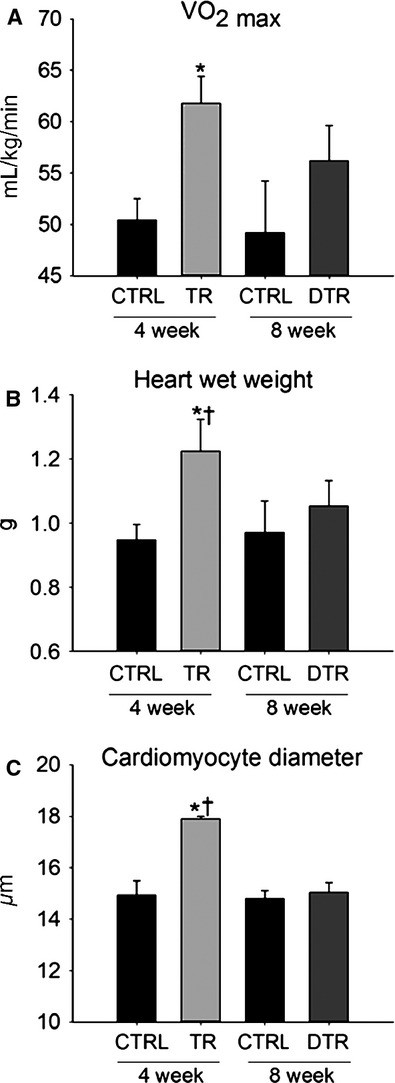
Exercise-induced improvements in VO_2max_, myocardial mass and cardiomyocyte hypertrophy are lost following detraining. A–C, VO_2max_ (A), heart weight (B) and LV cardiomyocyte diameter (C) are increased following 4 weeks of exercise training but return to CTRL levels after detraining. Data are Mean ± SD, *P < 0.05 vs. 4 week and 8 week CTRL, †*P* < 0.05 vs. DTR; *n* = 6 per group.

Following 4 weeks of intensity-controlled treadmill exercise training, VO_2max_ had increased significantly above (11.34 mL kg^−1^ min^−1^) CTRL (Fig.1A, *P* = 0.001). However, following 4 weeks of detraining, this adaptation was reduced with VO_2max_ being only 6.99 mL kg^−1^ min^−1^ above, but not significantly different to CTRL. The increase in aerobic exercise capacity seen with training was accompanied by positive anatomical and physiological cardiac remodeling. Cardiac mass was significantly greater in TR animals (∼29% greater) than CTRL (*P* < 0.001), but this regressed to ∼8% above CTRL in DTR animals (Fig.1B).

Echocardiography revealed that increased cardiac mass was due at least in part to LV remodeling as LV mass was also ∼32% greater in TR animals than CTRL (*P* < 0.001). The exercise trained group showed a significant thickening of the interventricular septum (SWT, 15% increase) and LV posterior wall (PWT, 15% increase), with an increased diastolic and reduced systolic internal LV diameter (Table1). These anatomical changes were accompanied by significant increases in both ejection fraction (EF, by 10%) and fractional shortening (FS, by 10%), compared to CTRL (*P* ≤ 0.001) (Table1). However, following a 4 week period of detraining, these exercise-induced improvements were almost completely lost with mean LV mass, SWT, PWT, EF, and FS declining to levels not significantly different to CTRL (Table1).

**Table 1:** Echocardiographic data from animals after 4 weeks of training (TR) and followed by 4 weeks of detraining (DTR) compared to CTRL

Criteria	4 week CTRL	TR	8 week CTRL	DTR
SWTD (mm)	1.92 ± 0.12	2.22 ± 0.07*^,^†	1.94 ± 0.10	2.01 ± 0.06
SWTS (mm)	3.04 ± 0.18	3.55 ± 0.19*^,^†	3.07 ± 0.19	3.15 ± 0.22
LVEDD (mm)	6.45 ± 0.30	6.78 ± 0.12	6.44 ± 0.32	6.56 ± 0.13
LVESD (mm)	3.43 ± 0.14	2.92 ± 0.14*^,^†	3.41 ± 0.29	3.34 ± 0.21
PWTD (mm)	1.89 ± 0.21	2.15 ± 0.10*	1.89 ± 0.16	1.98 ± 0.06
PWTS (mm)	2.85 ± 0.34	3.27 ± 0.10*	2.84 ± 0.37	2.98 ± 0.08
Fractional shortening (%)	46.84 ± 1.92	56.99 ± 1.64*^,^†	47.01 ± 3.55	49.09 ± 3.83
Ejection fraction (%)	71.71 ± 2.03	81.48 ± 1.40*^,^†	71.82 ± 3.78	73.97 ± 3.88
LV mass (mg)	844.62 ± 90.17	1116.58 ± 42.52*^,^†	846.96 ± 73.02	927.64 ± 43.12

SWTD, septal wall thickness in diastole; SWTS, septal wall thickness in systole; LVEDD, left ventricle end-diastolic diameter; LVESD, left ventricle end-systolic diameter; PWTD, posterior wall thickness in diastole; PWTS, posterior wall thickness in systole (data are mean+SD, ^*^P < 0.05 vs. 4 and 8 week CTRL, †*P* < 0.05 vs. DTR; *n* = 6 for all groups).

These functional and physiological data were corroborated at the cellular level as cardiomyocyte hypertrophy measured by LV cardiomyocyte diameter was 20% greater in TR animals than CTRL (Fig.1C, *P* ≤ 0.001), however, following detraining the hypertrophic response to exercise was lost with average cardiomyocyte diameter similar to CTRL (Fig.1C).

### Stimulation for exercise-induced new cardiomyocyte and capillary formation is lost upon cessation of training

**Figure 2:**
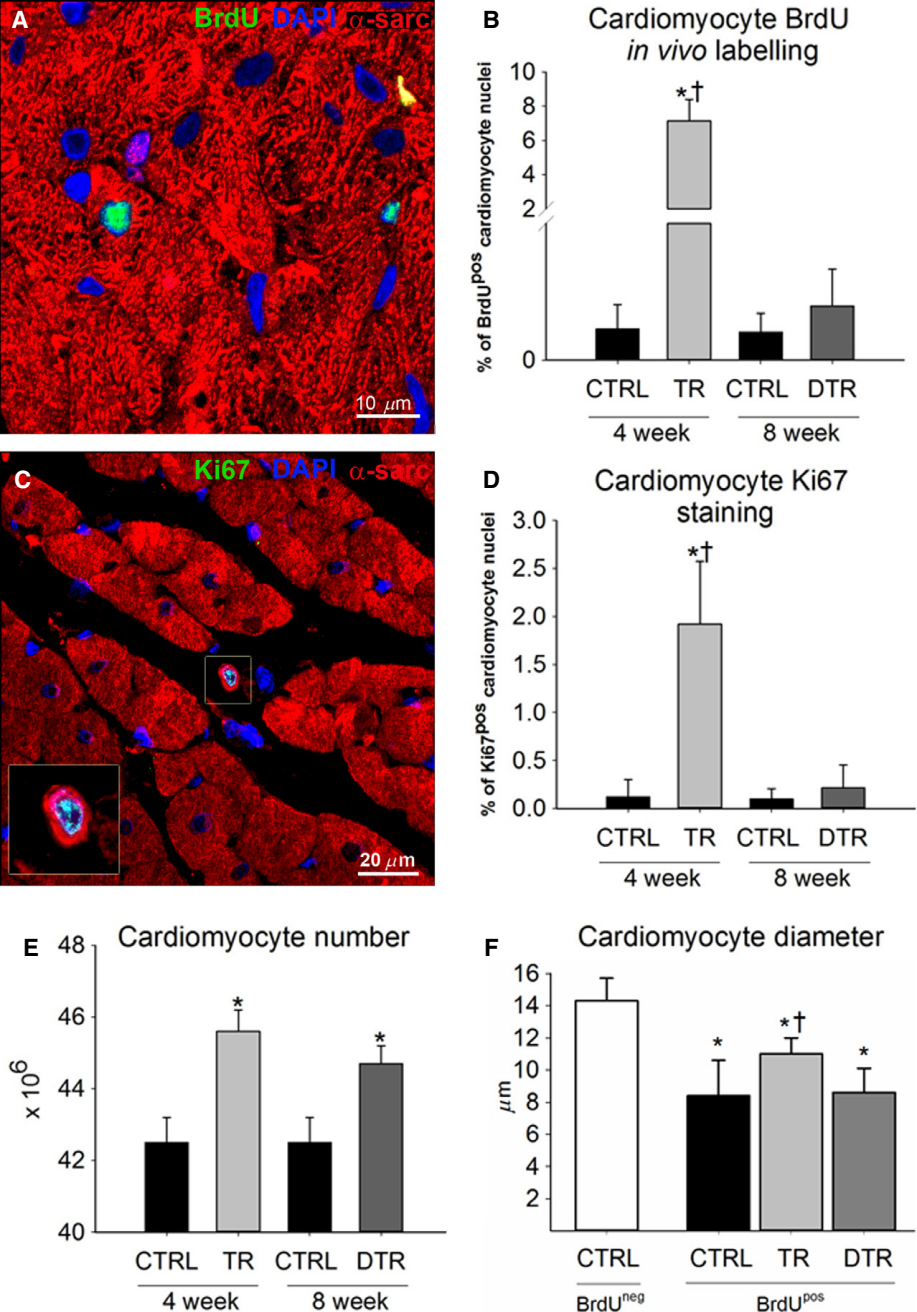
Cardiac myogenesis is induced following a period of intensity-controlled treadmill exercise training but not during detraining. A, Representative image of a small newly formed BrdU^pos^ (green) cardiomyocyte (*α*-sarcomeric actin; red) in the LV of a TR animal. Nuclei detected by DAPI in blue. B, The % number of BrdU^pos^ cardiomyocytes formed during exercise training and detraining. C, Representative image of a small newly formed Ki67^pos^ (green) cardiomyocyte in the LV of a TR animal. (Inset is ×2 zoom of boxed area). D, The % number of Ki67^pos^ cardiomyocytes formed during exercise training and detraining. E, Total cardiomyocyte number increases with exercise and remains elevated following detraining. *P < 0.05 vs. 4 week and 8 week CTRL, †*P* < 0.05 vs. DTR. F, BrdU^pos^ cardiomyocyte size following exercise training and detraining. *P < 0.05 vs. CTRL BrdU-negative cardiomyocyte, †*P* < 0.05 vs. CTRL and DTR BrdU-positive cardiomyocyte. Data are Mean ± SD, *n* = 6 per group.

To directly monitor new cardiomyocyte formation we injected BrdU to CTRL, TR, and DTR animals. TR animals received BrdU during the 4 week training period only to identify cardiomyocytes formed as a result of exercise training, whereas animals in the DTR group were administered BrdU in the 4 weeks following training cessation to track new cardiomyocyte formation during the detraining period only. To determine the normal level of myogenesis, CTRL animals also received BrdU for a 4 week period. Double staining for BrdU/*α*-sarcomeric actin revealed the presence of small, newly formed, mononucleated, BrdU^pos^ cardiomyocytes in the LV of TR animals (Fig.2A). Comparable with our previous findings (Waring et al. 2014), after 4 weeks of exercise training, ∼7% of cardiomyocytes were BrdU^pos^ (Fig.2B). New cardiomyocyte formation was also detected in CTRL animals, but at a rate significantly lower than in TR rats (Fig.2B, *P* < 0.001). Upon cessation of training, the stimulus for new cardiomyocyte formation was lost with a similar number of BrdU^pos^ cardiomyocytes observed in the LV of DTR (∼0.4%) and CTRL (∼0.2%) animals (Fig.2B).

Exercise-induced new cardiomyocyte formation was further confirmed by the presence of a subpopulation of cycling (Ki67^pos^) cardiomyocytes (Fig.2C–D), with a significantly higher incidence in the LV of TR animals (∼2%, *P* < 0.001). Corroborating the BrdU data, detraining of the animals did not lead to an increase in new cycling Ki67^pos^ cardiomyocyte formation, with DTR animals (∼0.2%) having a similar percentage of Ki67^pos^ cardiomyocytes as CTRL (∼0.1%; Fig.2D). BrdU labeling in vivo provided an accumulative measure of new cardiomyocyte formation over the exercise training and then the detraining protocol, whereas the Ki67^pos^ cardiomyocytes are those that were still or had recently been in the cell cycle just prior to sacrifice. Therefore, it is not surprising that the number of BrdU^pos^ cardiomyocytes is higher than that of Ki67^pos^ cardiomyocytes.

The increase in the number of newly formed BrdU^pos^ and Ki67^pos^ cardiomyocytes infers an increase in cardiomyocyte number with exercise training. Indeed, the total number of ventricular cardiomyocytes increased significantly (∼7%) with exercise training (Fig.2E, *P* < 0.001), agreeing with the percent increase in the number of newly formed BrdU^pos^ cardiomyocytes (Fig.2B). A period of detraining led to no further increase in cardiomyocyte number, with DTR animals having a ∼5% increase in cardiomyocyte number, compared to CTRL animals (Fig.2E, *P* < 0.001). These data show that animals had retained the new cardiomyocytes formed as a result of exercise training.

The size of the BrdU^pos^ cardiomyocytes over 28 days corroborated their newly formed status in that they were significantly smaller than the BrdU-negative myocytes in the same hearts (Fig.2F). Interestingly, BrdU^pos^ cardiomyocytes in exercised hearts were significantly larger than BrdU^pos^ cardiomyocytes in CTRL and DTR hearts (Fig.2F), showing that exercise training has a hypertrophic effect on newly formed cardiomyocytes.

**Figure 3:**
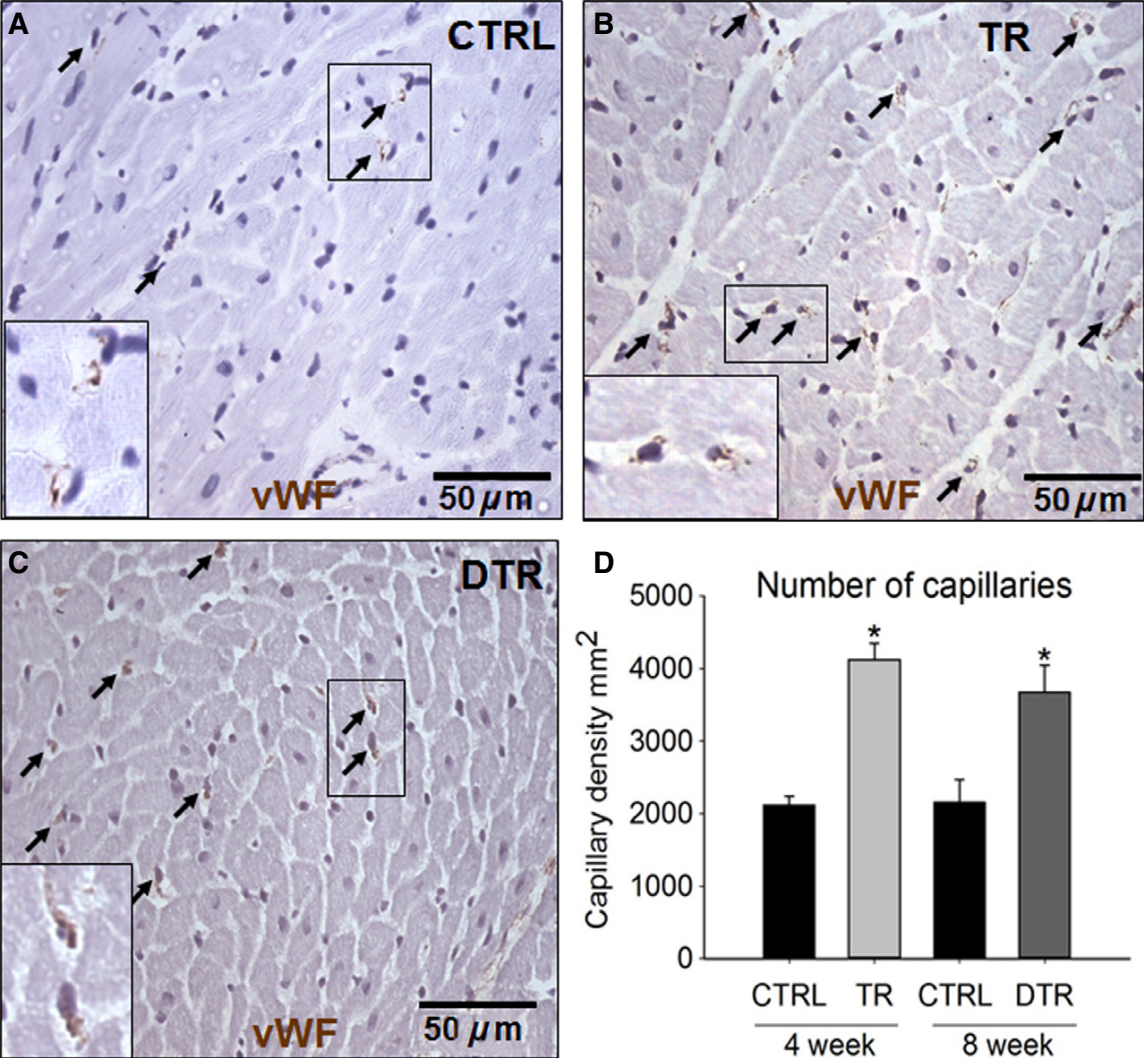
Intensity-controlled treadmill exercise-induced angiogenesis and capillary density remains elevated during detraining. A-C, Representative images of vWF (brown) capillaries (arrows) identified in the LV of CTRL (A), TR (B) and DTR (C) animals (Insets are ×2 zoom of boxed areas). Nuclei detected by hematoxylin in purple. D, Capillary density in the LV of CTRL, TR and DTR animals. Data are Mean ± SD, *P < 0.05 vs. 4 week and 8 week CTRL; *n* = 6 per group.

In conjunction with new cardiomyocyte formation and number, capillary density was also significantly elevated following exercise training (4123 ± 227 per mm^2^) compared to CTRL (2117 ± 118 mm^2^, *P* ≤ 0.001) and remained significantly elevated over CTRL but did not continue to increase following the detraining period (3673 ± 374 per mm^2^), with a similar number of capillaries in both TR and DTR animals (Fig.3A–D). These findings suggest that the exercise-induced increase in angiogenesis and new cardiomyocyte formation and number that occurred during training stopped upon cessation of exercise (Figs.2 and 3).

### Cessation of exercise training leads to diminished eCSC activation

**Figure 4:**
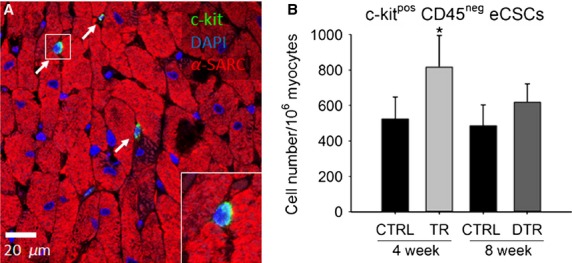
c-kit^pos^ eCSCs activation following exercise training and detraining. A, Representative image showing c-kit^pos^ (green) eCSCs (arrows) from the LV (*α*-sarcomeric actin; red) of a TR animal (Inset is ×2 zoom of boxed area). Nuclei detected by DAPI in blue. B, c-kit^pos^CD45^neg^ eCSC number in the LV of CTRL, TR and DTR animals. Data are Mean ± SD, *P < 0.05 vs. all. *n* = 6 per group.

We have previously demonstrated a precursor–product relationship between c-kit^pos^ eCSC activation and proliferation, and newly formed, differentiated cardiac cell progeny with intensity-controlled exercise training (Waring et al. 2014) and following diffuse myocardial injury (Ellison et al. 2013). With the lack of new cardiomyocyte and capillary formation with detraining reported here, we hypothesized that the stimulus for eCSC activation would also be removed. Indeed, the number of c-kit^pos^, CD45^neg^ eCSCs seen after detraining (640 ± 98) was significantly (*P* < 0.05) decreased than at the end of the training period (884 ± 112), and comparable to CTRL animals (482 ± 132; Fig.4, *P* = 0.269).

## Discussion

The results presented here show upon cessation of intensity-controlled treadmill running exercise training, the stimulus for c-kit^pos^ eCSC activation, new cardiomyocyte and capillary formation is lost, as is the hypertrophic response of the cardiomyocytes. Indeed, detraining leads to regression of the exercise-induced anatomical and functional benefits with LV mass and wall thickness, cardiac function, and maximal exercise capacity returning to levels similar to their CTRL counterparts.

### Detraining leads to lack of eCSC activation and cardiomyocyte and capillary hyperplasia

The degree of new cardiomyocyte formation (BrdU^pos^ and Ki67^pos^ cardiomyocytes) with exercise training reported here closely matches what we and others found previously (Boström et al. 2010; Waring et al. 2014). The lack of new cardiomyocyte and capillary formation during the detraining period was accompanied by a reduction in c-kit^pos^ CD45^neg^ eCSC activation, with levels in DTR animals similar to CTRL. We previously showed that with exercise training there is an increase in key growth factors in the myocardium which act as activating factors on c-kit^pos^ eCSCs, stimulating their multiplication and differentiation toward the cardiomyocyte and endothelial lineage (Waring et al. 2014). The present findings suggest that this growth factor upregulation is rapidly lost with detraining, concomitant with a return of the eCSCs to quiescence and normal cardiac homeostasis. That just 4-weeks of detraining can revert the exercise-induced cellular and physiological adaptations to levels of CTRL has important implications for physical inactivity as a major risk factor for heart disease and failure.

The source of newly formed cardiomyocytes is currently hotly debated, with evidence that a very small number of new, cycling cardiomyocytes are the product of preexisting cardiomyocytes (Senyo et al. 2013). Other evidence suggests that new cardiomyocytes predominantly originate from resident endogenous cardiac stem/progenitor cells (Hsieh et al. 2007; Loffredo et al. 2011; Ellison et al. 2013). We and others have previously shown through lineage tracing that c-kit^pos^ eCSCs give rise to new cardiomyocytes in vivo (Ellison et al. 2013; van Berlo et al. 2014), and that a precursor–product relationship exists between eCSC activation and new cardiomyocyte formation following exercise training (Waring et al. 2014).

The continued elevation of cardiomyocyte number and capillary density following detraining shown here indicates that despite the loss of the hypertrophic and hyperplasic stimuli, cessation of training does not stimulate/initiate a program of myocardial cell loss. Interestingly, we also show that exercise training is a hypertrophic stimulus for the newly formed BrdU-positive cardiomyocytes, which leads to improved maturation. Enhancing maturation of stem-cell-derived cardiomyocytes is an important, ongoing strategy in regenerative medicine and cardiology.

The degree to which capillary density was increased following exercise seen here is in agreement with that of other groups (Efthimiadou et al. 2004, 2006; Marini et al. 2008). Furthermore, previous studies have reported much lower increases (10–20%) in capillary density following exercise training (Iemitsu et al. 2006; Leosco et al. 2008), however, these were in much older animals suggesting that the intensity of the response to exercise declines with age. In agreement with the present findings, Marini et al. (2008) also reported that increased capillary density gained through exercise training persisted but did not continue to increase during a detraining period, showing a small but nonsignificant decline in capillary density similar to our own. The small decline in capillary density after detraining may be due to some capillaries contributing/fusing to increase arteriole size and number (White et al. 1998). Whether the number of new cardiomyocytes and capillaries formed through exercise training, decline further following a longer period of detraining and the mechanism that governs this remains to be ascertained.

### Cessation of exercise quickly leads to regression of exercise-induced anatomical and functional adaptations

The regression toward CTRL levels occurs quickly, as we found that almost 50% of the exercise-induced increase in VO_2max_ was lost after 4 weeks of detraining. Kemi et al. (2004) reported that 50% of the exercise gained improvements in VO_2max_ were lost at 2 weeks and continued to regress to 5% above CTRL at 4 weeks of detraining, following a 10 week program of high intensity interval training in female Sprague–Dawley rats (Kemi et al. 2004). The variation in the rate of loss of exercise-induced improvements of VO_2max_ may reflect differences in the exercise training protocols (continuous running for 30 min day^−1^, 4 days week^−1^ for 4 weeks in this study vs interval training for 1 h day^−1^, 5 days week^−1^ for 10 weeks) or between different species and/or sex of rat.

In addition to improvements in VO_2max_ and cardiomyocyte hypertrophy, exercise training enhanced cardiac mass and function. Increased cardiac mass seen after exercise training was a result of a significant thickening of the interventricular septum and the LV posterior wall, adaptations well documented in the rat and human heart following endurance exercise (Pluim et al. 2000; Baggish et al. 2008; Belabbas et al. 2008; Bocalini et al. 2010; Spence et al. 2011; Waring et al. 2014). This increase in wall thickness was accompanied by a dilation of the LV in diastole, consistent with our previous findings (Waring et al. 2014) and other studies (Jin et al. 2000; Pluim et al. 2000; Baggish et al. 2008; Belabbas et al. 2008; Bocalini et al. 2010; Weiner et al. 2012). In systole, LV diameter was significantly reduced in trained animals compared to CTRL, consistent with our previous findings (Waring et al. 2014). Unlike LVEDD, the effect of endurance exercise on LVESD is less defined with some groups similar to us reporting a decrease (Jin et al. 2000; Spence et al. 2011), whereas others document an increase with endurance exercise (Baggish et al. 2008; Bocalini et al. 2010). In these latter studies, unlike our own, EF and FS were not significantly enhanced with exercise despite significant increases in cardiac mass, LV wall thickness and significant improvements in other measures of systolic function such as LV end-systolic volume, stroke volume, and cardiac output. A static or even reduced EF in exercised subjects has been attributed as a secondary effect of LV dilation and has been shown to have a high prevalence among elite distance runners (Legaz Arrese et al. 2005). Baggish et al. (2008) suggested that LV EF has limitations as a measure of LV systolic function given that it is not able to account for geometric changes in chamber architecture.

The LV anatomical adaptation following detraining is currently ill defined. Following detraining, we found that anatomical and functional adaptations gained through exercise were predominantly lost (Table1), in agreement with previous findings (Bocalini et al. 2010). Reports in humans show persistence in LV cavity dilation despite a complete reduction in LV mass and complete regression of LV wall thickness, following detraining (Pelliccia et al. 2002; Spence et al. 2011; Weiner et al. 2012). Pelliccia et al. (2002) found that the persistence in LV dilation after detraining was associated with an increase in body weight and persistent recreational physical activity, which could provide a stimulus for increased LV cavity size. Spence et al. (2011) found that 6 months of supervised, intensive, endurance exercise training in young, untrained subjects lead to increased aerobic fitness, LV mass, LVEDV, and interventricular wall thickness, which after 6 weeks of detraining had all returned to baseline values except LVEDV which remained elevated (Spence et al. 2011). These findings raise the hypothesis that following a period of detraining changes in wall thickness occur before those in cavity dimension.

Finally, it remains to be determined what happens to the myocardium during a second period of training & detraining, does the heart adapt more rapidly indicating that cells retain memory of the previous training response? Additionally, given the cellular response to exercise could be diminished with age and it appears that less physiological benefit is gained with age (Iemitsu et al. 2006; Leosco et al. 2008), is the response by which these adaptations are lost, greater in an aged population? Moreover, given the response documented here refers to a high intensity exercise program, would exercise-induced adaptations gained from a lower intensity exercise training protocol be lost as rapidly given the degree to which the stimulus is removed is far less? Work to address these questions is now in progress.
